# Bis(μ-hexa­deca­noato-κ^2^ *O*:*O*)bis­[(2,2′-bipyridine-κ^2^ *N*,*N*′)(hexa­deca­noato-κ*O*)copper(II)] methanol disolvate

**DOI:** 10.1107/S1600536811013559

**Published:** 2011-04-16

**Authors:** Ahmad Nazeer Che Mat, Norbani Abdullah, Hamid Khaledi, Jia Ti Tee

**Affiliations:** a Department of Chemistry, University of Malaya, 50603 Kuala Lumpur, Malaysia

## Abstract

The asymmetric unit of the title compound, [Cu_2_(C_16_H_31_O_2_)_4_(C_10_H_8_N_2_)_2_]·2CH_3_OH, contains one half-mol­ecule of the metal complex solvated by a methanol mol­ecule. In the complex, two of the metal atoms are doubly bridged by two monodentate bridging hexa­deca­noate ligands around a center of inversion. The square-pyramidal geometry around each Cu^II^ ion is completed by a terminal hexa­deca­noate O atom and two N atoms from a 2,2′-bipyridine ligand. The alkyl chains of the carboxyl­ate ligands are arranged in a parallel manner with an all-*trans* conformation. In the crystal, a π–π inter­action formed by the bipyridine rings [centroid–centroid separation = 3.7723 (17) Å] and inter­molecular C—H⋯O hydrogen bonds link the complex mol­ecules into infinite chains along the *b* axis. An O—H⋯O interaction between the methanol solvate and one of the carboxylate O atoms is also observed.

## Related literature

For background to metallomesogens, see: Giroud-Godquin (1998 ▶). For the structures of similar copper(II) complexes, see: Antolini *et al.* (1985 ▶); Zhang *et al.* (2006 ▶). For a description of the geometry of complexes with a five-coordinate metal atom, see: Addison *et al.* (1984 ▶).
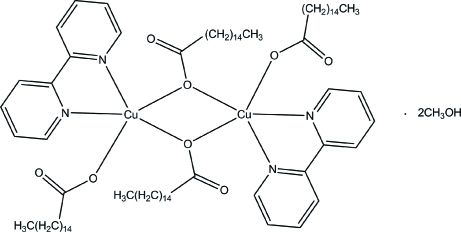


## Experimental

### 

#### Crystal data

[Cu_2_(C_16_H_31_O_2_)_4_(C_10_H_8_N_2_)_2_]·2CH_4_O
*M* *_r_* = 1525.16
Triclinic, 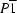

*a* = 9.6064 (3) Å
*b* = 9.7506 (3) Å
*c* = 24.0234 (8) Å
α = 92.559 (2)°
β = 98.681 (2)°
γ = 95.516 (2)°
*V* = 2210.14 (12) Å^3^
*Z* = 1
Mo *K*α radiation
μ = 0.54 mm^−1^
*T* = 296 K
0.40 × 0.27 × 0.09 mm

#### Data collection

Bruker APEXII CCD diffractometer
Absorption correction: multi-scan (*SADABS*; Sheldrick, 1996 ▶) *T*_min_ = 0.814, *T*_max_ = 0.953
13457 measured reflections
8088 independent reflections
5498 reflections with *I* > 2σ(*I*)
*R*_int_ = 0.034

#### Refinement

*R*[*F* ^2^ > 2σ(*F* ^2^)] = 0.050
*wR*(*F* ^2^) = 0.127
*S* = 0.99
8088 reflections
466 parameters
1 restraint
H atoms treated by a mixture of independent and constrained refinement
Δρ_max_ = 0.31 e Å^−3^
Δρ_min_ = −0.24 e Å^−3^

### 

Data collection: *APEX2* (Bruker, 2007 ▶); cell refinement: *SAINT* (Bruker, 2007 ▶); data reduction: *SAINT*; program(s) used to solve structure: *SHELXS97* (Sheldrick, 2008 ▶); program(s) used to refine structure: *SHELXL97* (Sheldrick, 2008 ▶); molecular graphics: *X-SEED* (Barbour, 2001 ▶); software used to prepare material for publication: *SHELXL97* and *publCIF* (Westrip, 2010 ▶).

## Supplementary Material

Crystal structure: contains datablocks I, global. DOI: 10.1107/S1600536811013559/om2420sup1.cif

Structure factors: contains datablocks I. DOI: 10.1107/S1600536811013559/om2420Isup2.hkl

Additional supplementary materials:  crystallographic information; 3D view; checkCIF report

## Figures and Tables

**Table 1:** Hydrogen-bond geometry (Å, °)

*D*—H⋯*A*	*D*—H	H⋯*A*	*D*⋯*A*	*D*—H⋯*A*
C33—H33⋯O1^i^	0.93	2.50	3.093 (3)	122
C33—H33⋯O3^i^	0.93	2.57	3.071 (3)	114
C35—H35⋯O2^ii^	0.93	2.42	3.129 (3)	134
C39—H39⋯O4^iii^	0.93	2.41	3.258 (4)	152
C41—H41⋯O5^iv^	0.93	2.46	3.153 (4)	131
C42—H42⋯O1	0.93	2.59	3.096 (3)	115
O5—H5⋯O2	0.86 (2)	1.89 (3)	2.732 (4)	166 (6)

Symmetry codes: (i) 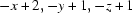; (ii) 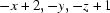; (iii) 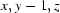; (iv) 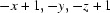.

**Fig. 1.**
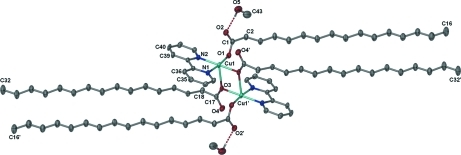
Thermal ellipsoid plot of the title compound at the 30% probability level. Atom labels for consecutively numbered rings and alkyl chains, as well as C-bound hydrogen atoms have been omitted for clarity. Symmetry code: ' = -x+2, –y+1, -z+1.

**Fig. 2.**
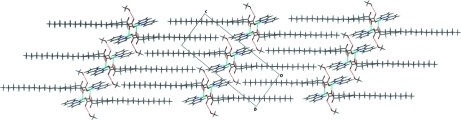
Packing view looking down the crystallographic a axis.

## supplementary crystallographic information

### Comment

Metallomesogens are metal containing liquid crystals. Active research in this field started around 30 years ago (Giroud-Godquin, 1998), and these materials have found useful applications as ordered solvents, catalysts, and templates for synthesis, optical and ferroelectric systems, electronic or ionic conductors, and membranes. However, most metallomesogens have high melting points (greater than 523 K), high viscosity, and narrow isotropic range. Our research group is focused on functional low-temperature and thermally stable metallomesogens for spintronic, electronic, photonic and catalytic applications. To achieve this, we applied the concept of symmetry reduction and use of ligands with long (linear or branched) alkyl chains. Herein, we report the crystal structure of one such complex.

The title compound is a centrosymmetric dinuclear copper(II) complex in which the metal ions are five-coordinate in a square-pyramidal geometry with the τ value (Addison *et al.*, 1984) of 0.016. The coordination geometry around each metal center is defined by two nitrogen atoms from a 2,2'-bipyridine and two O atoms from two monodentate hexadecanoato ligands at the basal positions. The square-pyramidal coordination of Cu(II) is completed by bonding to the bridging hexadecanoate O atom in the axial direction with Cu—O distance of 2.349 (2) Å. Within this double bridged dimer, the Cu···Cu distance [3.3740 (6) Å] is comparable to those observed in similar structures (Antolini *et al.*, 1985; Zhang *et al.*, 2006). The alkyl chains of the carboxylate ligands are arranged in a parallel manner with an all *trans* conformation. The methanol solvent molecule is a hydrogen bond donor to the carboxylate O2 atom. In the crystal, intermolecular C—H···O interactions (Table 1) connect the molecules into infinite chains along the *b* axis. The one-dimensional link is supplemented by a π-π stacking interaction between the anti-parallely arranged bipyridine rings of the adjacent molecules, the pyridyl rings centroid-centroid separation being 3.7723 (17) Å. Intramolecular C—H···O interactions are also observed.

### Experimental

An aqueous solution of copper(II)nitrate trihydrate (5.7 g, 23.4 mmol) was added portionwise to a hot ethanolic solution (150 ml) of hexadecanoic acid (6 g, 23.4 mmol) and *p*-aminobenzoic acid (3.2 g, 23.6 mmol). The green solution formed was allowed to cool to room temperature, and then an excess amount of ammonia (30%) was added. The purple solution formed was stirred at room temperature overnight, and then heated gently to remove excess ammonia and get the pale blue precipitate of [Cu_2_(*p*-H_2_NC_6_H_4_COO)_2_(CH_3_(CH_2_)_14_COO)_2_]. 2,2'-Bipyridine (0.19 g, 1.2 mmol) was added to a suspension of [Cu_2_(*p*-H_2_NC_6_H_4_COO)_2_(CH_3_(CH_2_)_14_COO)_2_] (1 g, 1.1 mmol) in a 1:2 mixture of methanol-ethanol (60 ml). The mixture was heated for 30 minutes and the black precipitate was filtered off. The small blue crystals obtained from the filtrate, on standing overnight, were recrystallized from methanol-THF (1:1), to give the dark blue crystals of the title compound after two weeks.

### Refinement

The C-bound hydrogen atoms were placed at calculated positions (C–H 0.93–0.97 Å), and were treated as riding on their parent carbon atoms. The oxygen-bound H atom was located in a difference Fourier map and refined with distance restraint of O–H 0.82±0.02 Å. For hydrogen atoms U*iso*(H) were set to 1.2–1.5 times U*eq*(carrier atom).

### Crystal data

**Table:**

[Cu_2_(C_16_H_31_O_2_)_4_(C_10_H_8_N_2_)_2_]·2CH_4_O	*Z* = 1
*M**_r_* = 1525.16	*F*(000) = 830
Triclinic, *P*1	*D*_x_ = 1.146 Mg m^−^^3^
Hall symbol: -P 1	Mo *K*α radiation, λ = 0.71073 Å
*a* = 9.6064 (3) Å	Cell parameters from 2321 reflections
*b* = 9.7506 (3) Å	θ = 2.5–21.5°
*c* = 24.0234 (8) Å	µ = 0.54 mm^−^^1^
α = 92.559 (2)°	*T* = 296 K
β = 98.681 (2)°	Block, blue
γ = 95.516 (2)°	0.40 × 0.27 × 0.09 mm
*V* = 2210.14 (12) Å^3^	

### Data collection

**Table:**

Bruker APEXII CCD diffractometer	8088 independent reflections
Radiation source: fine-focus sealed tube	5498 reflections with *I* > 2σ(*I*)
graphite	*R*_int_ = 0.034
φ and ω scans	θ_max_ = 25.5°, θ_min_ = 1.7°
Absorption correction: multi-scan (*SADABS*; Sheldrick, 1996)	*h* = −11→11
*T*_min_ = 0.814, *T*_max_ = 0.953	*k* = −11→11
13457 measured reflections	*l* = −29→29

### Refinement

**Table:**

Refinement on *F*^2^	Primary atom site location: structure-invariant direct methods
Least-squares matrix: full	Secondary atom site location: difference Fourier map
*R*[*F*^2^ > 2σ(*F*^2^)] = 0.050	Hydrogen site location: inferred from neighbouring sites
*wR*(*F*^2^) = 0.127	H atoms treated by a mixture of independent and constrained refinement
*S* = 0.99	*w* = 1/[σ^2^(*F*_o_^2^) + (0.061*P*)^2^] where *P* = (*F*_o_^2^ + 2*F*_c_^2^)/3
8088 reflections	(Δ/σ)_max_ = 0.011
466 parameters	Δρ_max_ = 0.31 e Å^−^^3^
1 restraint	Δρ_min_ = −0.24 e Å^−^^3^

### Special details

**Table:**

Geometry. All e.s.d.'s (except the e.s.d. in the dihedral angle between two l.s. planes) are estimated using the full covariance matrix. The cell e.s.d.'s are taken into account individually in the estimation of e.s.d.'s in distances, angles and torsion angles; correlations between e.s.d.'s in cell parameters are only used when they are defined by crystal symmetry. An approximate (isotropic) treatment of cell e.s.d.'s is used for estimating e.s.d.'s involving l.s. planes.
Refinement. Refinement of *F*^2^ against ALL reflections. The weighted *R*-factor *wR* and goodness of fit *S* are based on *F*^2^, conventional *R*-factors *R* are based on *F*, with *F* set to zero for negative *F*^2^. The threshold expression of *F*^2^ > σ(*F*^2^) is used only for calculating *R*-factors(gt) *etc*. and is not relevant to the choice of reflections for refinement. *R*-factors based on *F*^2^ are statistically about twice as large as those based on *F*, and *R*- factors based on ALL data will be even larger.

### Fractional atomic coordinates and isotropic or equivalent isotropic displacement parameters (Å^2^)

**Table:**

	*x*	*y*	*z*	*U*_iso_*/*U*_eq_	
Cu1	0.91189 (3)	0.34121 (3)	0.498126 (15)	0.03823 (13)	
O1	0.7446 (2)	0.42240 (19)	0.51491 (9)	0.0475 (5)	
O2	0.6702 (2)	0.2457 (2)	0.56137 (10)	0.0619 (6)	
O3	0.96731 (19)	0.53048 (18)	0.44537 (8)	0.0426 (5)	
O4	0.9778 (2)	0.7002 (2)	0.38786 (10)	0.0602 (6)	
N1	1.0740 (2)	0.2359 (2)	0.48224 (10)	0.0378 (6)	
N2	0.8058 (2)	0.1916 (2)	0.44362 (10)	0.0394 (6)	
C1	0.6751 (3)	0.3687 (3)	0.55073 (14)	0.0452 (8)	
C2	0.5984 (3)	0.4670 (3)	0.58304 (14)	0.0542 (9)	
H2A	0.5558	0.5314	0.5577	0.065*	
H2B	0.5236	0.4154	0.5987	0.065*	
C3	0.7025 (3)	0.5457 (3)	0.63028 (14)	0.0556 (9)	
H3A	0.7496	0.4796	0.6534	0.067*	
H3B	0.7741	0.5998	0.6138	0.067*	
C4	0.6373 (3)	0.6407 (3)	0.66775 (14)	0.0606 (9)	
H4A	0.5932	0.7091	0.6450	0.073*	
H4B	0.5635	0.5874	0.6834	0.073*	
C5	0.7410 (4)	0.7138 (4)	0.71538 (15)	0.0671 (10)	
H5A	0.8128	0.7689	0.6993	0.081*	
H5B	0.7878	0.6448	0.7369	0.081*	
C6	0.6812 (4)	0.8064 (4)	0.75564 (15)	0.0641 (10)	
H6A	0.6378	0.8781	0.7347	0.077*	
H6B	0.6072	0.7525	0.7709	0.077*	
C7	0.7877 (4)	0.8737 (4)	0.80388 (15)	0.0657 (10)	
H7A	0.8621	0.9264	0.7884	0.079*	
H7B	0.8304	0.8017	0.8249	0.079*	
C8	0.7305 (4)	0.9680 (4)	0.84449 (15)	0.0666 (10)	
H8A	0.6861	1.0392	0.8235	0.080*	
H8B	0.6576	0.9150	0.8607	0.080*	
C9	0.8383 (4)	1.0361 (4)	0.89138 (15)	0.0695 (11)	
H9A	0.9104	1.0896	0.8749	0.083*	
H9B	0.8837	0.9645	0.9117	0.083*	
C10	0.7853 (4)	1.1292 (4)	0.93316 (15)	0.0681 (10)	
H10A	0.7387	1.2000	0.9127	0.082*	
H10B	0.7140	1.0753	0.9500	0.082*	
C11	0.8932 (4)	1.1989 (4)	0.97973 (16)	0.0720 (11)	
H11A	0.9389	1.1283	1.0006	0.086*	
H11B	0.9650	1.2522	0.9630	0.086*	
C12	0.8389 (4)	1.2925 (4)	1.02059 (15)	0.0724 (11)	
H12A	0.7682	1.2386	1.0377	0.087*	
H12B	0.7914	1.3617	0.9995	0.087*	
C13	0.9456 (4)	1.3652 (4)	1.06694 (16)	0.0772 (11)	
H13A	0.9927	1.2960	1.0882	0.093*	
H13B	1.0167	1.4188	1.0499	0.093*	
C14	0.8910 (4)	1.4587 (4)	1.10716 (16)	0.0781 (12)	
H14A	0.8196	1.4050	1.1240	0.094*	
H14B	0.8439	1.5277	1.0857	0.094*	
C15	0.9958 (5)	1.5325 (5)	1.15394 (19)	0.0965 (14)	
H15A	1.0422	1.4641	1.1760	0.116*	
H15B	1.0678	1.5861	1.1374	0.116*	
C16	0.9367 (5)	1.6263 (5)	1.19290 (19)	0.1150 (18)	
H16A	0.8961	1.6985	1.1722	0.172*	
H16B	1.0110	1.6659	1.2221	0.172*	
H16C	0.8649	1.5748	1.2095	0.172*	
C17	0.9304 (3)	0.5849 (3)	0.39814 (13)	0.0421 (7)	
C18	0.8202 (3)	0.4979 (3)	0.35562 (13)	0.0512 (8)	
H18A	0.7488	0.4538	0.3754	0.061*	
H18B	0.7739	0.5582	0.3293	0.061*	
C19	0.8802 (3)	0.3880 (3)	0.32266 (13)	0.0482 (8)	
H19A	0.9534	0.4315	0.3036	0.058*	
H19B	0.9238	0.3257	0.3487	0.058*	
C20	0.7695 (3)	0.3054 (3)	0.27940 (14)	0.0565 (9)	
H20A	0.6993	0.2584	0.2990	0.068*	
H20B	0.7219	0.3691	0.2551	0.068*	
C21	0.8245 (3)	0.1999 (3)	0.24292 (14)	0.0590 (9)	
H21A	0.8656	0.1318	0.2668	0.071*	
H21B	0.8994	0.2455	0.2253	0.071*	
C22	0.7130 (4)	0.1261 (4)	0.19725 (15)	0.0657 (10)	
H22A	0.6424	0.0743	0.2151	0.079*	
H22B	0.6660	0.1948	0.1756	0.079*	
C23	0.7670 (4)	0.0293 (4)	0.15724 (15)	0.0678 (10)	
H23A	0.8096	−0.0422	0.1786	0.081*	
H23B	0.8410	0.0800	0.1408	0.081*	
C24	0.6571 (4)	−0.0390 (4)	0.10995 (15)	0.0671 (10)	
H24A	0.5851	−0.0930	0.1264	0.081*	
H24B	0.6117	0.0324	0.0895	0.081*	
C25	0.7126 (4)	−0.1317 (4)	0.06851 (15)	0.0679 (10)	
H25A	0.7834	−0.0772	0.0517	0.081*	
H25B	0.7598	−0.2019	0.0891	0.081*	
C26	0.6041 (4)	−0.2025 (4)	0.02180 (15)	0.0679 (10)	
H26A	0.5341	−0.2584	0.0385	0.081*	
H26B	0.5558	−0.1325	0.0015	0.081*	
C27	0.6611 (4)	−0.2929 (4)	−0.01978 (15)	0.0679 (10)	
H27A	0.7115	−0.3611	0.0008	0.082*	
H27B	0.7295	−0.2363	−0.0370	0.082*	
C28	0.5540 (4)	−0.3672 (4)	−0.06597 (15)	0.0697 (10)	
H28A	0.4855	−0.4239	−0.0488	0.084*	
H28B	0.5037	−0.2991	−0.0867	0.084*	
C29	0.6122 (4)	−0.4565 (4)	−0.10674 (15)	0.0720 (11)	
H29A	0.6636	−0.5234	−0.0857	0.086*	
H29B	0.6802	−0.3992	−0.1239	0.086*	
C30	0.5078 (4)	−0.5336 (4)	−0.15314 (16)	0.0751 (11)	
H30A	0.4400	−0.5910	−0.1359	0.090*	
H30B	0.4561	−0.4667	−0.1741	0.090*	
C31	0.5658 (5)	−0.6224 (4)	−0.19391 (17)	0.0912 (14)	
H31A	0.6188	−0.6884	−0.1730	0.109*	
H31B	0.6322	−0.5647	−0.2117	0.109*	
C32	0.4606 (5)	−0.7003 (5)	−0.23923 (18)	0.1087 (17)	
H32A	0.4007	−0.7659	−0.2226	0.163*	
H32B	0.5096	−0.7480	−0.2649	0.163*	
H32C	0.4039	−0.6370	−0.2593	0.163*	
C33	1.2115 (3)	0.2658 (3)	0.50422 (13)	0.0452 (8)	
H33	1.2389	0.3473	0.5264	0.054*	
C34	1.3123 (3)	0.1815 (3)	0.49531 (14)	0.0481 (8)	
H34	1.4065	0.2064	0.5108	0.058*	
C35	1.2743 (3)	0.0604 (3)	0.46349 (14)	0.0495 (8)	
H35	1.3415	0.0007	0.4579	0.059*	
C36	1.1338 (3)	0.0283 (3)	0.43977 (13)	0.0462 (8)	
H36	1.1054	−0.0529	0.4175	0.055*	
C37	1.0366 (3)	0.1174 (3)	0.44938 (12)	0.0374 (7)	
C38	0.8836 (3)	0.0946 (3)	0.42662 (12)	0.0367 (7)	
C39	0.8228 (3)	−0.0172 (3)	0.39013 (13)	0.0497 (8)	
H39	0.8778	−0.0836	0.3787	0.060*	
C40	0.6794 (4)	−0.0272 (3)	0.37133 (14)	0.0572 (9)	
H40	0.6364	−0.1009	0.3468	0.069*	
C41	0.5997 (3)	0.0712 (3)	0.38858 (14)	0.0539 (9)	
H41	0.5027	0.0649	0.3761	0.065*	
C42	0.6662 (3)	0.1794 (3)	0.42469 (13)	0.0456 (8)	
H42	0.6124	0.2464	0.4364	0.055*	
O5	0.6666 (4)	0.1341 (4)	0.66351 (13)	0.1153 (12)	
H5	0.656 (7)	0.175 (5)	0.6327 (15)	0.173*	
C43	0.7910 (5)	0.1699 (5)	0.6985 (2)	0.1218 (19)	
H43A	0.8472	0.0934	0.7000	0.183*	
H43B	0.7724	0.1940	0.7356	0.183*	
H43C	0.8415	0.2475	0.6845	0.183*	

### Atomic displacement parameters (Å^2^)

**Table:**

	*U*^11^	*U*^22^	*U*^33^	*U*^12^	*U*^13^	*U*^23^
Cu1	0.0363 (2)	0.0308 (2)	0.0444 (2)	0.00502 (14)	−0.00033 (16)	−0.01253 (15)
O1	0.0442 (11)	0.0392 (11)	0.0593 (15)	0.0086 (9)	0.0092 (11)	−0.0083 (11)
O2	0.0750 (15)	0.0396 (13)	0.0710 (17)	0.0061 (11)	0.0141 (13)	−0.0066 (11)
O3	0.0505 (12)	0.0330 (10)	0.0406 (12)	0.0066 (9)	−0.0032 (10)	−0.0084 (9)
O4	0.0753 (15)	0.0399 (12)	0.0614 (15)	0.0001 (12)	0.0018 (12)	0.0020 (11)
N1	0.0379 (12)	0.0287 (12)	0.0446 (15)	0.0048 (10)	0.0013 (11)	−0.0066 (11)
N2	0.0396 (13)	0.0326 (12)	0.0444 (15)	0.0048 (10)	0.0029 (12)	−0.0072 (11)
C1	0.0363 (16)	0.0461 (19)	0.049 (2)	0.0046 (14)	−0.0028 (15)	−0.0146 (16)
C2	0.0430 (17)	0.0542 (19)	0.063 (2)	0.0080 (15)	0.0056 (16)	−0.0197 (17)
C3	0.0507 (18)	0.0547 (19)	0.057 (2)	0.0079 (16)	0.0008 (17)	−0.0182 (17)
C4	0.059 (2)	0.064 (2)	0.055 (2)	0.0191 (17)	−0.0021 (17)	−0.0208 (18)
C5	0.066 (2)	0.070 (2)	0.063 (2)	0.0134 (19)	0.0036 (19)	−0.0224 (19)
C6	0.063 (2)	0.069 (2)	0.058 (2)	0.0150 (18)	0.0021 (18)	−0.0217 (18)
C7	0.068 (2)	0.068 (2)	0.060 (2)	0.0147 (19)	0.0034 (19)	−0.0177 (19)
C8	0.069 (2)	0.073 (2)	0.055 (2)	0.0106 (19)	0.0043 (19)	−0.0191 (19)
C9	0.068 (2)	0.075 (2)	0.062 (2)	0.009 (2)	0.004 (2)	−0.021 (2)
C10	0.070 (2)	0.075 (2)	0.056 (2)	0.008 (2)	0.0067 (19)	−0.0178 (19)
C11	0.067 (2)	0.078 (3)	0.065 (3)	0.003 (2)	0.001 (2)	−0.021 (2)
C12	0.076 (2)	0.075 (2)	0.061 (2)	0.010 (2)	0.000 (2)	−0.024 (2)
C13	0.077 (3)	0.079 (3)	0.069 (3)	0.003 (2)	0.001 (2)	−0.020 (2)
C14	0.081 (3)	0.082 (3)	0.063 (3)	0.008 (2)	−0.007 (2)	−0.019 (2)
C15	0.092 (3)	0.106 (3)	0.079 (3)	−0.002 (3)	−0.006 (3)	−0.028 (3)
C16	0.140 (4)	0.109 (4)	0.084 (3)	0.004 (3)	−0.004 (3)	−0.039 (3)
C17	0.0434 (16)	0.0392 (17)	0.0420 (19)	0.0115 (14)	−0.0002 (15)	−0.0090 (15)
C18	0.0495 (18)	0.0500 (18)	0.049 (2)	0.0106 (15)	−0.0102 (15)	−0.0089 (15)
C19	0.0520 (18)	0.0460 (17)	0.0429 (19)	0.0037 (15)	0.0000 (15)	−0.0081 (15)
C20	0.059 (2)	0.0553 (19)	0.049 (2)	−0.0006 (16)	−0.0012 (17)	−0.0160 (16)
C21	0.0565 (19)	0.061 (2)	0.053 (2)	0.0002 (17)	−0.0026 (17)	−0.0195 (17)
C22	0.064 (2)	0.065 (2)	0.062 (2)	−0.0002 (18)	0.0034 (19)	−0.0245 (19)
C23	0.064 (2)	0.072 (2)	0.060 (2)	0.0040 (19)	−0.0010 (19)	−0.0249 (19)
C24	0.066 (2)	0.068 (2)	0.061 (2)	−0.0003 (19)	0.0018 (19)	−0.0249 (19)
C25	0.068 (2)	0.071 (2)	0.059 (2)	0.0068 (19)	−0.0021 (19)	−0.0239 (19)
C26	0.070 (2)	0.070 (2)	0.057 (2)	0.0007 (19)	0.0009 (19)	−0.0244 (19)
C27	0.071 (2)	0.071 (2)	0.056 (2)	0.0050 (19)	0.0004 (19)	−0.0215 (19)
C28	0.074 (2)	0.071 (2)	0.057 (2)	0.002 (2)	0.0013 (19)	−0.0211 (19)
C29	0.075 (2)	0.075 (2)	0.059 (2)	0.010 (2)	−0.005 (2)	−0.023 (2)
C30	0.082 (3)	0.078 (3)	0.058 (2)	0.005 (2)	−0.004 (2)	−0.020 (2)
C31	0.102 (3)	0.094 (3)	0.069 (3)	0.016 (3)	−0.006 (2)	−0.036 (2)
C32	0.115 (4)	0.118 (4)	0.080 (3)	0.013 (3)	−0.015 (3)	−0.047 (3)
C33	0.0416 (16)	0.0331 (15)	0.058 (2)	0.0040 (13)	0.0018 (15)	−0.0085 (14)
C34	0.0368 (16)	0.0427 (17)	0.064 (2)	0.0066 (14)	0.0046 (16)	−0.0007 (16)
C35	0.0483 (18)	0.0391 (17)	0.065 (2)	0.0141 (14)	0.0161 (16)	−0.0040 (16)
C36	0.0509 (18)	0.0346 (16)	0.053 (2)	0.0048 (14)	0.0114 (16)	−0.0117 (14)
C37	0.0434 (16)	0.0287 (14)	0.0398 (17)	0.0027 (12)	0.0075 (14)	−0.0034 (13)
C38	0.0441 (16)	0.0300 (14)	0.0345 (16)	0.0012 (12)	0.0036 (13)	−0.0024 (12)
C39	0.0553 (19)	0.0392 (17)	0.050 (2)	0.0023 (15)	0.0021 (16)	−0.0167 (15)
C40	0.065 (2)	0.0416 (18)	0.055 (2)	−0.0066 (16)	−0.0075 (17)	−0.0188 (16)
C41	0.0457 (17)	0.0539 (19)	0.053 (2)	−0.0041 (16)	−0.0114 (16)	−0.0064 (16)
C42	0.0405 (16)	0.0432 (17)	0.051 (2)	0.0041 (14)	0.0019 (15)	−0.0063 (15)
O5	0.116 (2)	0.135 (3)	0.078 (2)	−0.052 (2)	0.003 (2)	0.003 (2)
C43	0.120 (4)	0.148 (5)	0.085 (4)	−0.033 (4)	0.003 (3)	0.012 (3)

### Geometric parameters (Å, °)

**Table:**

Cu1—O1	1.945 (2)	C19—C20	1.512 (4)
Cu1—O3^i^	1.9617 (17)	C19—H19A	0.9700
Cu1—N2	2.005 (2)	C19—H19B	0.9700
Cu1—N1	2.019 (2)	C20—C21	1.507 (4)
Cu1—O3	2.349 (2)	C20—H20A	0.9700
O1—C1	1.268 (4)	C20—H20B	0.9700
O2—C1	1.235 (4)	C21—C22	1.515 (4)
O3—C17	1.289 (3)	C21—H21A	0.9700
O3—Cu1^i^	1.9617 (17)	C21—H21B	0.9700
O4—C17	1.222 (3)	C22—C23	1.499 (4)
N1—C33	1.346 (3)	C22—H22A	0.9700
N1—C37	1.357 (3)	C22—H22B	0.9700
N2—C42	1.342 (3)	C23—C24	1.511 (4)
N2—C38	1.347 (3)	C23—H23A	0.9700
C1—C2	1.519 (4)	C23—H23B	0.9700
C2—C3	1.519 (4)	C24—C25	1.505 (4)
C2—H2A	0.9700	C24—H24A	0.9700
C2—H2B	0.9700	C24—H24B	0.9700
C3—C4	1.506 (4)	C25—C26	1.504 (4)
C3—H3A	0.9700	C25—H25A	0.9700
C3—H3B	0.9700	C25—H25B	0.9700
C4—C5	1.502 (4)	C26—C27	1.502 (4)
C4—H4A	0.9700	C26—H26A	0.9700
C4—H4B	0.9700	C26—H26B	0.9700
C5—C6	1.507 (4)	C27—C28	1.502 (4)
C5—H5A	0.9700	C27—H27A	0.9700
C5—H5B	0.9700	C27—H27B	0.9700
C6—C7	1.505 (4)	C28—C29	1.487 (5)
C6—H6A	0.9700	C28—H28A	0.9700
C6—H6B	0.9700	C28—H28B	0.9700
C7—C8	1.510 (4)	C29—C30	1.502 (4)
C7—H7A	0.9700	C29—H29A	0.9700
C7—H7B	0.9700	C29—H29B	0.9700
C8—C9	1.493 (4)	C30—C31	1.484 (5)
C8—H8A	0.9700	C30—H30A	0.9700
C8—H8B	0.9700	C30—H30B	0.9700
C9—C10	1.501 (4)	C31—C32	1.493 (5)
C9—H9A	0.9700	C31—H31A	0.9700
C9—H9B	0.9700	C31—H31B	0.9700
C10—C11	1.495 (4)	C32—H32A	0.9600
C10—H10A	0.9700	C32—H32B	0.9600
C10—H10B	0.9700	C32—H32C	0.9600
C11—C12	1.494 (4)	C33—C34	1.363 (4)
C11—H11A	0.9700	C33—H33	0.9300
C11—H11B	0.9700	C34—C35	1.365 (4)
C12—C13	1.496 (4)	C34—H34	0.9300
C12—H12A	0.9700	C35—C36	1.384 (4)
C12—H12B	0.9700	C35—H35	0.9300
C13—C14	1.486 (5)	C36—C37	1.372 (4)
C13—H13A	0.9700	C36—H36	0.9300
C13—H13B	0.9700	C37—C38	1.481 (4)
C14—C15	1.497 (5)	C38—C39	1.391 (3)
C14—H14A	0.9700	C39—C40	1.377 (4)
C14—H14B	0.9700	C39—H39	0.9300
C15—C16	1.490 (5)	C40—C41	1.371 (4)
C15—H15A	0.9700	C40—H40	0.9300
C15—H15B	0.9700	C41—C42	1.377 (4)
C16—H16A	0.9600	C41—H41	0.9300
C16—H16B	0.9600	C42—H42	0.9300
C16—H16C	0.9600	O5—C43	1.359 (5)
C17—C18	1.519 (4)	O5—H5	0.86 (2)
C18—C19	1.513 (4)	C43—H43A	0.9600
C18—H18A	0.9700	C43—H43B	0.9600
C18—H18B	0.9700	C43—H43C	0.9600
			
O1—Cu1—O3^i^	90.51 (8)	C20—C19—C18	113.0 (2)
O1—Cu1—N2	95.52 (9)	C20—C19—H19A	109.0
O3^i^—Cu1—N2	172.47 (8)	C18—C19—H19A	109.0
O1—Cu1—N1	173.45 (9)	C20—C19—H19B	109.0
O3^i^—Cu1—N1	93.43 (8)	C18—C19—H19B	109.0
N2—Cu1—N1	80.17 (9)	H19A—C19—H19B	107.8
O1—Cu1—O3	89.86 (8)	C21—C20—C19	115.3 (3)
O3^i^—Cu1—O3	77.36 (8)	C21—C20—H20A	108.4
N2—Cu1—O3	107.08 (8)	C19—C20—H20A	108.4
N1—Cu1—O3	96.10 (8)	C21—C20—H20B	108.4
C1—O1—Cu1	119.20 (19)	C19—C20—H20B	108.4
C17—O3—Cu1^i^	113.34 (17)	H20A—C20—H20B	107.5
C17—O3—Cu1	142.73 (18)	C20—C21—C22	114.1 (3)
Cu1^i^—O3—Cu1	102.64 (8)	C20—C21—H21A	108.7
C33—N1—C37	117.7 (2)	C22—C21—H21A	108.7
C33—N1—Cu1	126.72 (18)	C20—C21—H21B	108.7
C37—N1—Cu1	115.38 (17)	C22—C21—H21B	108.7
C42—N2—C38	118.8 (2)	H21A—C21—H21B	107.6
C42—N2—Cu1	125.58 (19)	C23—C22—C21	115.3 (3)
C38—N2—Cu1	115.63 (17)	C23—C22—H22A	108.4
O2—C1—O1	124.4 (3)	C21—C22—H22A	108.4
O2—C1—C2	119.8 (3)	C23—C22—H22B	108.4
O1—C1—C2	115.8 (3)	C21—C22—H22B	108.4
C3—C2—C1	109.7 (2)	H22A—C22—H22B	107.5
C3—C2—H2A	109.7	C22—C23—C24	115.4 (3)
C1—C2—H2A	109.7	C22—C23—H23A	108.4
C3—C2—H2B	109.7	C24—C23—H23A	108.4
C1—C2—H2B	109.7	C22—C23—H23B	108.4
H2A—C2—H2B	108.2	C24—C23—H23B	108.4
C4—C3—C2	114.7 (3)	H23A—C23—H23B	107.5
C4—C3—H3A	108.6	C25—C24—C23	115.2 (3)
C2—C3—H3A	108.6	C25—C24—H24A	108.5
C4—C3—H3B	108.6	C23—C24—H24A	108.5
C2—C3—H3B	108.6	C25—C24—H24B	108.5
H3A—C3—H3B	107.6	C23—C24—H24B	108.5
C5—C4—C3	113.9 (3)	H24A—C24—H24B	107.5
C5—C4—H4A	108.8	C26—C25—C24	115.9 (3)
C3—C4—H4A	108.8	C26—C25—H25A	108.3
C5—C4—H4B	108.8	C24—C25—H25A	108.3
C3—C4—H4B	108.8	C26—C25—H25B	108.3
H4A—C4—H4B	107.7	C24—C25—H25B	108.3
C4—C5—C6	116.4 (3)	H25A—C25—H25B	107.4
C4—C5—H5A	108.2	C27—C26—C25	115.3 (3)
C6—C5—H5A	108.2	C27—C26—H26A	108.4
C4—C5—H5B	108.2	C25—C26—H26A	108.4
C6—C5—H5B	108.2	C27—C26—H26B	108.4
H5A—C5—H5B	107.3	C25—C26—H26B	108.4
C7—C6—C5	114.7 (3)	H26A—C26—H26B	107.5
C7—C6—H6A	108.6	C26—C27—C28	116.2 (3)
C5—C6—H6A	108.6	C26—C27—H27A	108.2
C7—C6—H6B	108.6	C28—C27—H27A	108.2
C5—C6—H6B	108.6	C26—C27—H27B	108.2
H6A—C6—H6B	107.6	C28—C27—H27B	108.2
C6—C7—C8	115.7 (3)	H27A—C27—H27B	107.4
C6—C7—H7A	108.4	C29—C28—C27	115.5 (3)
C8—C7—H7A	108.4	C29—C28—H28A	108.4
C6—C7—H7B	108.4	C27—C28—H28A	108.4
C8—C7—H7B	108.4	C29—C28—H28B	108.4
H7A—C7—H7B	107.4	C27—C28—H28B	108.4
C9—C8—C7	114.9 (3)	H28A—C28—H28B	107.5
C9—C8—H8A	108.5	C28—C29—C30	116.9 (3)
C7—C8—H8A	108.5	C28—C29—H29A	108.1
C9—C8—H8B	108.5	C30—C29—H29A	108.1
C7—C8—H8B	108.5	C28—C29—H29B	108.1
H8A—C8—H8B	107.5	C30—C29—H29B	108.1
C8—C9—C10	116.6 (3)	H29A—C29—H29B	107.3
C8—C9—H9A	108.2	C31—C30—C29	116.9 (3)
C10—C9—H9A	108.2	C31—C30—H30A	108.1
C8—C9—H9B	108.2	C29—C30—H30A	108.1
C10—C9—H9B	108.2	C31—C30—H30B	108.1
H9A—C9—H9B	107.3	C29—C30—H30B	108.1
C11—C10—C9	116.7 (3)	H30A—C30—H30B	107.3
C11—C10—H10A	108.1	C30—C31—C32	116.4 (4)
C9—C10—H10A	108.1	C30—C31—H31A	108.2
C11—C10—H10B	108.1	C32—C31—H31A	108.2
C9—C10—H10B	108.1	C30—C31—H31B	108.2
H10A—C10—H10B	107.3	C32—C31—H31B	108.2
C12—C11—C10	116.0 (3)	H31A—C31—H31B	107.3
C12—C11—H11A	108.3	C31—C32—H32A	109.5
C10—C11—H11A	108.3	C31—C32—H32B	109.5
C12—C11—H11B	108.3	H32A—C32—H32B	109.5
C10—C11—H11B	108.3	C31—C32—H32C	109.5
H11A—C11—H11B	107.4	H32A—C32—H32C	109.5
C11—C12—C13	116.9 (3)	H32B—C32—H32C	109.5
C11—C12—H12A	108.1	N1—C33—C34	122.7 (3)
C13—C12—H12A	108.1	N1—C33—H33	118.6
C11—C12—H12B	108.1	C34—C33—H33	118.6
C13—C12—H12B	108.1	C33—C34—C35	119.7 (3)
H12A—C12—H12B	107.3	C33—C34—H34	120.2
C14—C13—C12	116.5 (3)	C35—C34—H34	120.2
C14—C13—H13A	108.2	C34—C35—C36	118.6 (3)
C12—C13—H13A	108.2	C34—C35—H35	120.7
C14—C13—H13B	108.2	C36—C35—H35	120.7
C12—C13—H13B	108.2	C37—C36—C35	119.4 (3)
H13A—C13—H13B	107.3	C37—C36—H36	120.3
C13—C14—C15	117.5 (3)	C35—C36—H36	120.3
C13—C14—H14A	107.9	N1—C37—C36	121.8 (2)
C15—C14—H14A	107.9	N1—C37—C38	113.6 (2)
C13—C14—H14B	107.9	C36—C37—C38	124.6 (2)
C15—C14—H14B	107.9	N2—C38—C39	121.7 (3)
H14A—C14—H14B	107.2	N2—C38—C37	114.8 (2)
C16—C15—C14	115.7 (4)	C39—C38—C37	123.4 (3)
C16—C15—H15A	108.4	C40—C39—C38	118.3 (3)
C14—C15—H15A	108.4	C40—C39—H39	120.8
C16—C15—H15B	108.4	C38—C39—H39	120.8
C14—C15—H15B	108.4	C41—C40—C39	120.2 (3)
H15A—C15—H15B	107.4	C41—C40—H40	119.9
C15—C16—H16A	109.5	C39—C40—H40	119.9
C15—C16—H16B	109.5	C40—C41—C42	118.6 (3)
H16A—C16—H16B	109.5	C40—C41—H41	120.7
C15—C16—H16C	109.5	C42—C41—H41	120.7
H16A—C16—H16C	109.5	N2—C42—C41	122.4 (3)
H16B—C16—H16C	109.5	N2—C42—H42	118.8
O4—C17—O3	123.0 (3)	C41—C42—H42	118.8
O4—C17—C18	121.1 (3)	C43—O5—H5	116 (4)
O3—C17—C18	115.9 (3)	O5—C43—H43A	109.5
C19—C18—C17	113.9 (2)	O5—C43—H43B	109.5
C19—C18—H18A	108.8	H43A—C43—H43B	109.5
C17—C18—H18A	108.8	O5—C43—H43C	109.5
C19—C18—H18B	108.8	H43A—C43—H43C	109.5
C17—C18—H18B	108.8	H43B—C43—H43C	109.5
H18A—C18—H18B	107.7		

Symmetry codes: (i) −*x*+2, −*y*+1, −*z*+1.

### Hydrogen-bond geometry (Å, °)

**Table:**

*D*—H···*A*	*D*—H	H···*A*	*D*···*A*	*D*—H···*A*
C33—H33···O1^i^	0.93	2.50	3.093 (3)	122
C33—H33···O3^i^	0.93	2.57	3.071 (3)	114
C35—H35···O2^ii^	0.93	2.42	3.129 (3)	134
C39—H39···O4^iii^	0.93	2.41	3.258 (4)	152
C41—H41···O5^iv^	0.93	2.46	3.153 (4)	131
C42—H42···O1	0.93	2.59	3.096 (3)	115
O5—H5···O2	0.86 (2)	1.89 (3)	2.732 (4)	166 (6)

Symmetry codes: (i) −*x*+2, −*y*+1, −*z*+1; (ii) −*x*+2, −*y*, −*z*+1; (iii) *x*, *y*−1, *z*; (iv) −*x*+1, −*y*, −*z*+1.
